# Battling 21st-Century Scourges with a 14th-Century Toolbox^1^

**DOI:** 10.3201/eid1011.040797_12

**Published:** 2004-11

**Authors:** Martin Cetron, Pattie Simone

**Affiliations:** * Centers for Disease Control and Prevention, Atlanta, Georgia, USA

**Keywords:** quarantine approaches, SARS, SARS outbreaks, quarantine measures

A range of quarantine approaches were used in five jurisdictions heavily affected by the outbreak of severe acute respiratory syndrome (SARS) in 2003. Implementation of modern quarantine was resource intensive, involved coordination of multiple sectors of society, frequently required new legislative actions or authorities, and was highly dependent on effective communication.

In Toronto, Ontario, Canada, quarantine ranged from home quarantine with active surveillance to enhanced passive surveillance augmented by education about prevention and a contact number to call if symptoms developed. Healthcare workers were occasionally required to adhere to "work quarantine." New legislation in Ontario authorized compulsory quarantine with active follow-up for compliance. Although 30,000 people in Toronto were recommended for quarantine, enforcement orders had to be issued in only 27 instances. A comprehensive infrastructure was developed to support those in quarantine; masks, thermometers, food, and financial assistance, as well as psychosocial support, were provided. Should SARS return to Toronto, the same measures would be used to ensure that close contacts of infected persons are isolated and actively monitored.

In Taiwan, from April 28 to July 4, 2003, travelers arriving from World Health Organization–designated SARS-affected areas were quarantined for 10 days (level B quarantine). During the SARS epidemic, 50,319 persons who were close contacts of SARS patients were placed under level A quarantine; suspected or probable SARS was diagnosed for 112 (0.22%). A total of 80,813 persons were placed under level B quarantine; 21 (0.03%) of these cases were diagnosed as suspected or probable SARS. The strategies were later modified as understanding of the infectivity of SARS increased, so that close contacts and travelers from local transmission areas were required to follow guidelines of self health management, including isolation at home only when they had a fever. Fever monitoring at international ports initially continued year-round; its ongoing utility will be further examined.

Singapore relied on effective quarantine of all persons who had unprotected close contact with symptomatic case-patients. Critical systems were implemented for quarantine policy and practice, legislative backing, communications, enforcement and surveillance, safeguards on public transport and hospital visits, financial support, operational costs, and compensation. As the gravity of the situation became clear, the Infectious Diseases Act was invoked to impose quarantine on exposed, potentially infectious persons. A Quarantine Board was set up to assist with decisions on a case-by-case basis. An important lesson was the value of clear communication. As part of a comprehensive financial and social support system, the government offered an allowance to self-employed persons to compensate for part of their lost income and to establishments with affected employees.

In Hong Kong, medical services were severely disrupted when 380 healthcare workers became ill with SARS. From April to June 2003, the economy lost an estimated U.S.$3 billion, gross domestic product growth fell by 3.7%, and exports slumped by 13.9%. SARS was controlled by a combination of measures, including disease surveillance, isolation of cases, heightened infection control, contact tracing, quarantine, entry and exit screening, and community engagement. Hospital isolation facilities and infection control training were strengthened by adding 1,000 extra isolation beds and a U.S.$20 million training fund. Retrospective analysis showed that SARS developed in 2.7% of household contacts in home quarantine, and approximately 90% of all case-patients had an identifiable epidemiologic link. Entry and exit screenings that use health declarations and temperature checks, which detected only two cases during the outbreak, have covered 90 million passengers, 5,000 of whom had fever. Addressing surge capacity was a key issue, in which the private medical sector and nongovernmental organizations proved pivotal in providing medical services, community education, and support for emergency operations, including quarantine both at home and at dedicated residential facilities.

Beijing, China, experienced the world's largest outbreak of SARS in spring 2003 with 2,521 reported probable cases. Quarantine played an important role in controlling the outbreak. By July 1, a total of 30,178 persons, 0.21% of the Beijing population, had been quarantined. Most close contacts were quarantined at home (60%); the rest were at designated sites, including hotels, universities, and construction worksites. In late April, fever checks were instituted at the airport, major train stations, and all 71 roads connecting Beijing to other areas; these sites used infrared thermometers to screen and axillary thermometers to confirm fever among passengers. As of June 30, 2003, of almost 14 million people screened, only 12 probable cases of SARS were identified. All healthcare workers in SARS-designated hospitals had to stay in designated hotels close to the hospitals rather than at home. After finishing their work with SARS patients, they were sent to resort areas for 2 more weeks. Top challenges for implementing quarantine included tracing contacts, maintaining movement restrictions even at home, and finding the resources to provide 10,000 people with supplies and psychological care. Nonetheless, the same quarantine measures will be implemented if SARS returns.

